# Diagnostic value of exosome-derived lncRNA PITPNA-AS1 in lung cancer

**DOI:** 10.3389/fimmu.2025.1539557

**Published:** 2025-04-24

**Authors:** Mujin Chen, XiaoHui Feng, ChengChen Liu, Yan Huang, LiJuan Su, XiaoFeng Li, JinFeng Zhu

**Affiliations:** ^1^ Department of Oncology, First Hospital of Quanzhou Affiliated to Fujian Medical University, Fujian, Quanzhou, China; ^2^ Department of Oncology, Loujiang New City Hospital of Taicang (Ruijin Hospital, Shanghai Jiao Tong University School of Medicine), Suzhou, China; ^3^ Department of Gastroenterology, WuWei City The Second People’s Hospital, Gansu, China

**Keywords:** lung cancer, exosomes, lncRNA PITPNA-AS1, FMR1, diagnosis

## Abstract

**Background:**

Lung cancer is one of the most lethal types of cancer, and effective diagnostic biomarkers are required. There is increasing evidences that exosome-secreted lncRNAs could play an important role in lung cancer diagnosis. However, the diagnostic value and molecular mechanism of the key lncRNA PITPNA-AS1 in lung cancer remain unclear.

**Methods:**

qRT-PCR was conducted to determine the levels of exosomal lncRNA PITPNA-AS1 in pleural effusions from lung adenocarcinoma, squamous cell lung carcinoma, and small cell lung cancer patients. Receiver operating characteristic (ROC) curve analyses were used to evaluate the diagnostic accuracy of PITPNA-AS1. Its role in lung cancer development was determined by a series of experiments, including CCK-8, flow cytometry, and transwell assays. RNA pull-down and RNA immunoprecipitation assays were carried out to examine the interaction between PITPNA-AS1 and Fragile X messenger ribonucleoprotein 1 (FMR1).

**Results:**

We discovered PITPNA-AS1 in exosomes from lung cancer patients. Its expression was significantly increased in lung cancer patients compared to non-cancer patients, and it was strongly associated with tumor stage, lymph node metastasis, and distant metastasis in all lung cancer subtypes assessed (all p<0.05). ROC curve analyses demonstrated that exosomal PITPNA-AS1 had a high accuracy for differentiating among lung cancer subtypes. Furthermore, PITPNA-AS1 boosted H1299 and A549 cell proliferation, migration, and invasion. Mechanistically, via direct interaction, PITPNA-AS1 increased FMR1 stability by preventing its ubiquitination.

**Conclusions:**

These results reveal that exosome-derived lncRNA PITPNA-AS1 acts as an oncogene to promote malignant biological behaviors and is a promising diagnostic biomarker in lung cancer.

## Introduction

With the increasing number of smokers and serious air pollution, lung cancer is the most common cause of cancer-related death worldwide (1, 2). Lung cancer is traditionally divided into small cell lung cancer (SCLC, approximately 15%) and non-small cell lung cancer (NSCLC, approximately 85%), of which lung adenocarcinoma (LUAD) and lung squamous cell carcinoma (LUSC) are the most common subtypes (3, 4). Despite continuous improvements in lung cancer treatment being made over many decades, only a small number of patients benefit from these therapies and 5-year survival rates are still below 20% (5). Due to the lack of specific clinical manifestations and rapid cancer progression, most patients with lung cancer are diagnosed at an advanced stage, which gains greater difficulties in the cancer therapy. Hence, it is important to identify biomarkers with high sensitivity and specificity for distinguish lung cancer from non-cancer conditions at an early stage.

Exosomes, 30–100 nm in diameter, act as bridges for intercellular communication by delivering messages in the form of proteins, lipids, and nucleic acids (mRNAs and noncoding RNAs) (6, 7). Recent discoveries regarding the tumor microenvironment in cancer patients have highlighted the relevance of exosomes in tumorigenesis and tumor progression (8–10). As tumors progress, the cargo in exosomes can be dynamically distributed throughout the body, and this cargo can be involved in tumor progression (11, 12). For instance, cancer cells can suppress immune functions by triggering apoptosis and exhaustion of various immune effector cells via the release of tumor-derived exosomal cargo, such as transforming growth factor (TGF)-β (13), FasL (14), and TRAIL (15). However, exosomal miR-BART6-3p has been shown to inhibit cancer cell metastasis and invasion by targeting LOC553103 (16). Thus, exosomes have gained increasing attention as critical elements in tumorigenesis and oncotherapy responses.

Long noncoding RNAs (lncRNAs) are typically more than 200 nucleotides in length and function as regulators of protein-coding genes (17). Notably, they can also be released from cells in exosomes and act as intercellular messengers. Recent researches have shown that exosomal lncRNAs can induce angiogenesis and regulate tumor cell apoptosis, proliferation, and migration (17, 18). For instance, the pituitary tumor-transforming 3 pseudogene (PTTG3P) is upregulated in hepatocellular carcinoma (HCC), functioning as an oncogene (19). Additionally, hypoxic exosomes participate in bladder tumor growth and development by secreting the lncRNA UCA1 (20). Thus, lncRNAs are emerging as potential diagnostic biomarkers in multiple cancers (21, 22).

Here, we investigated the diagnostic accuracy of lncRNA PITPNA-AS1 for differentiating among lung cancer subtypes, as well as its contribution to lung cancer progression and the underlying mechanism. Our results indicate that exosomal lncRNA PITPNA-AS1 is a promising biomarker for lung cancer diagnosis, and it may contribute to developing prognostic tools and therapeutic strategies for lung cancer.

## Materials and methods

### Patients and clinical samples

This study involved patients who were histopathologically diagnosed with lung cancer from February 2017 to February 2019 at Lishui Hospital of Zhejiang University. The exclusion criteria were as follows: malignant pleural effusions caused by lung metastasis; other cancers, systemic connective tissue disease, rheumatic disease, severe liver or kidney insufficiency, severe hypoproteinemia, severe heart failure; and leakage of lung cancer. Based on pathological stage classification according to the patients’ medical records, patients with SCLC were divided into stage I–II (15 cases) and III–IV (29 cases); patients with LUAD were divided into stage I–II (17 cases) and III–IV (31 cases), and patients with LUSC were divided into stage I–II (14 cases) and III–IV (26 cases). In addition, we enrolled non-cancer patients (i.e., free of cancer at enrollment) exhibiting massive pleural effusion (50 cases), comprising 13 cases of tuberculous pleurisy, 22 cases of pneumonia, 9 cases of lung abscess, and 6 cases of chest trauma. There were no significant differences in gender or age between the cancer and non-cancer patients (p >0.05). Patients demographic are presented in **Table 1**. The study was approved by the ethics committee of Lishui Hospital of Zhejiang University. All participants provided informed consent.

**Table 1:** Patient characteristics.

Characteristic	Benign Disease (n=50)	Lung Cancer		
		SCLC (n=44)	LUAD (n=48)	LUSC (n=40)
Age in years Median (range)	62 (57-67.25)	61.5 (57-64)	66.5 (60.25-71)	65 (59.25-68)
Gender				
Male	33 (66%)	29 (65.91%)	28 (58.33%)	14 (35%)
Female	17 (34%)	15 (34.09%)	20 (41.67%)	26 (65%)
Smoking status				
Yes	29 (58%)	26 (59.09%)	31 (64.58%)	25 (62.5%)
No	21 (42%)	18 (40.91%)	17 (35.42%)	15 (37.5%)
Stage				
I-II	/	15 (34.09)	17 (35.42%)	14 (35%)
III-IV	/	29 (55.91%)	31 (54.58%)	26 (65%)
Lymph metastasis				
Yes	/	31 (70.45%)	25 (50.83%)	28 (70%)
No	/	13 (28.55%)	13 (49.17%)	12 (30%)
Distal metastasis				
Yes	/	14 (31.82%)	9 (18.75%)	10 (25%)
No	/	30 (58.18%)	39 (81.25%)	30 (75%)

ECOG, Eastern cooperative oncology group; SCLC, small cell lung cancer; LUAD, lung adenocarcinoma; LUSC, lung squamous cell carcinoma.

### Exosome isolation and identification

Pleural effusions from lung cancer and non-cancer patients were ultra-centrifuged at 100,000 g for 120 min at 4°C. Next, the exosomes were purified according to a previously described standard protocol(29). The exosomes were observed by transmission electron microscopy (TEM; JEM-2100; Jeol, Japan). Nanoparticle tracking analysis (NTA) was performed using a Malvern Zetasizer Nano ZS90 system (Malvern Panalytical, Shanghai, China) to assess the concentration and size of the exosomes.

### Cell lines and culture

The human lung cancer cell lines H1299 and A549 were purchased from the Cell Bank of the Chinese Academy of Sciences (Shanghai, China). These cells were cultured at 37°C in 5% CO2 in Roswell Park Memorial Institute (RPMI) 1640 medium (Thermo Fisher Scientific, Inc., Waltham, MA, USA) supplemented with 10% fetal bovine serum (FBS, Biological Industries, Israel).

Plasmid construction and cell transfection. Specific siRNAs targeting lncRNA PITPNA-AS1 (si-PITPNA-AS1#1/2) and negative control siRNA (si-NC) were synthesized by GenePharma (Shanghai, China). H1299 and A549 cells were seeded in 6-well plates and transfected with the siRNAs (40 nM) using Lipofectamine 2000 (Invitrogen) following the manufacturer’s instructions. Thereafter, the cells were cultured for 24 h and then subjected to quantitative real-time (qRT)-PCR analysis. The target sequences were as follows: si-PITPNA-AS1#1: GCA GGG TGG ATA AAG AGG AAG ATG T; si-PITPNA-AS1#2: CCT CTT CCT AAT CCT GCC CTG GTA A; and si-NC: UGG UGA GAA AUU UAU UCA CAA A. Short hairpin RNAs (shRNA) specifically targeting PITPNA-AS1 and control shRNA were constructed by GenePharma (Shanghai, China). The cells were infected by lentivital-conditional medium to stably downregulated PITPNA-AS1 according to the manufacturers’ advice.

### Western blotting

A549 and H1299 cells were harvested using radioimmunoprecipitation assay (RIPA) buffer on ice for 30 min. Total protein was then extracted by centrifugation. Equal amounts of protein (20–40 μg) were subjected to 10% sodium dodecyl sulfate polyacrylamide gel electrophoresis (SDS-PAGE; Biosharp), transferred to 0.22-mm polyvinylidene fluoride (PVDF) membranes (Millipore, USA), and probed with primary antibodies (Abcam, Cambridge, UK) against the exosomal marker CD9 (ab92726, 1: 1000), the exosomal marker CD63 (ab134045, 1: 2000), Fragile X messenger ribonucleoprotein 1 (FMR1; ab17722, 1: 1000), or tubulin (ab6046, 1: 500) at 4°C overnight. After incubation with horseradish peroxidase-conjugated goat anti-mouse IgG H&L antibody (ab205719, 1: 10000; Abcam) for 2 h, the PVDF membranes were washed three times with phosphate-buffered saline and visualized using a chemiluminescence system. Glyceraldehyde 3-phosphate dehydrogenase (GAPDH; ab8245, 1: 10000) served as the internal control.

### RNA extraction and qRT-PCR

Total RNA was extracted from exosomes and A549 and H1299 cells using Trizol Reagent (Takara Bio Inc., Japan) according to the standard protocol. 1 μg mRNA was used for reverse transcription using a Prime Script RT Reagent Kit (Takara Bio Inc.). qRT-PCR was conducted with a Mastercycler^®^ pro (Eppendorf, Hamburg, Germany) using SYBR Green (Bio-Rad, China) using the following program: 94°C for 30 s and 40 cycles of 55°C for 1 min and 72°C for 30 s. GAPDH was used as the reference gene in parallel reactions. The primer sequences were shown in **Supplementary Table S1**.

### Cell proliferation assay

H1299 and A549 cells were seeded in each well of a 96-well plate and cultured overnight at 37°C with or without co-incubation with exosomes from lung cancer patients for 24 and 48 h. Thereafter, 10 µl CCK8 reagent was added for an extra 4 h of incubation. The absorbance (optical density) at 450 nm was then assessed using a microplate spectrophotometer (Thermo Fisher Scientific, Inc.).

### Apoptosis assay

Apoptosis was evaluated using an Annexin V-FITC Apoptosis Detection Kit (Beyotime Biotechnology) and flow cytometry following the manufacturer’s protocols. A549 and H1299 cells were cultured with or without co-incubation with exosomes. They were then incubated in binding buffer, followed by staining with Annexin V-FITC solution (200 μL) and PI solution (10 μL). Annexin Vstains the membranes of early-stage apoptotic cells (green) and PI stains the nuclei of advanced-stage apoptotic cells or necrotic cells (red). Data were analyzed using FlowJo software (Becton, Dickinson & Company).

### Transwell assay

To assess exosome function, A549 and H1299 cells were incubated with or without exosomes for 24 h. The cells were then suspended in FBS-free medium and seeded in 24-well transwell plates with inserts for migration assays. The inserts were free of serum, whereas the lower chamber contained 10% FBS. After 24 h, the cells on the lower surface of the membrane were fixed and stained according to the manufacturer’s instructions. Representative fields were photographed under a light microscope (200×) and the numbers of migrated cells in four random visual fields were counted. Each experiment was repeated three times.

### RNA immunoprecipitation assay

The interaction between lncRNA PITPNA-AS1 and RNA-binding protein FMR1 was assessed using an RIP assay kit according to the manufacturer’s instructions (Guangzhou Geneseed Biotechnology Co., Ltd, Guangzhou, China). Cell lysates were generated using complete RIP lysis buffer with DNase I and RNase A inhibitor and then cultured with protein A/G and 5 μg anti-PITPNA-AS1 antibody (Millipore) or nonspecific IgG (Millipore) at 4°C overnight. Thereafter, the cell lysates were eluted using washing buffer and elution buffer three times, respectively. Proteinase K was employed to isolate the bound RNAs, which were then subjected to qRT-PCR.

### RNA pull-down assay

RNA pull-down assay was performed to verify the interaction between lncRNA PITPNA-AS1 and FMR1 using an RNA pull-down assay kit according to the manufacturer’s instructions (Guangzhou Bersin Biotechnology Co., Ltd, Guangzhou, China). Cell lysates were incubated with biotinylated PITPNA-AS1 sense probe, PITPNA-AS1 anti-sense probe, or negative control. RNA–protein complexes were isolated using streptavidin agarose magnetic beads (Bersin Biotechnology). The level of FMR1 in the complexes was assessed by western blotting.

### Database analyses

The targets of PITPNA-AS1 were predicted using the RNA Interactome Database (www.rna-society.org/rnainter/home.html). Using the Protein Lysine Modification Database (PLMD, http://plmd.biocuckoo.org/), the lysine modification sites of FMR1 were assessed.

### Statistical analysis

The pathological results of biopsy or surgical specimens were used as the gold standard for diagnosis. Receiver operating characteristic (ROC) curve analyses were used to evaluate the diagnostic accuracy of lncRNA PITPNA-AS1. Area under the curve (AUC) >0.5 was considered to indicate diagnostic value. All data from triplicate assays are presented as mean ± SD. Student’s t-test was used to assess significant differences between pairs of groups. One-way analysis of variance (ANOVA) with Tukey’s test was used to assess significant differences among multiple groups. Two-tailed p<0.05 was considered to indicate significant differences.

## Results

**Figure 1:**
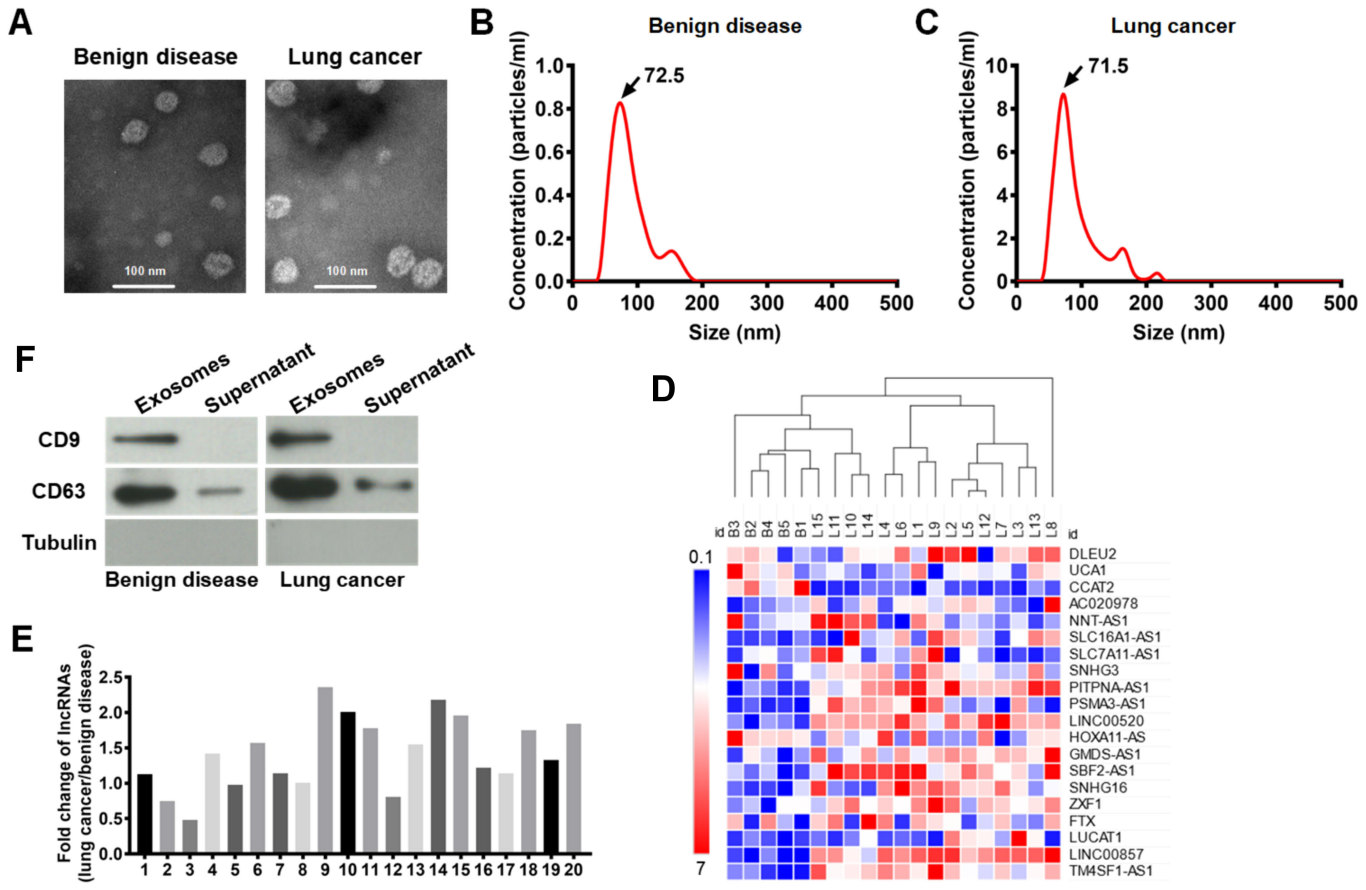
Exosomes were isolated from pleural effusions, and lncRNA PITPNA-AS1 was found to be enriched in the exosomes from lung cancer patients. **(A)** Representative transmisson electron microscopy images of exosomes from 15 lung cancers (5 per with SCLC, LUAD, LUSC) and 5 patients with benign disease. Scale bar: 100 nm. **(B, C)** Distribution of exosomes with diameters of 50–150 nm assessed using a Malvern Zetasizer Nano ZS90 system in benign disease group **(B)** and lung cancer group **(C)**. **(D)** Level of indicated proteins in exosomes and supernatant from 15 lung cancers and 5 patients with benign disease. **(E)** Fold changes of lncRNA expression. The expression of each lncRNA in benign disease group was normalized to 1. 1–20 indicates lncRNA DLEU2, lncRNA UCA1, lncRNA CCAT2, lncRNA-AC020978, lncRNA NNT-AS1, lncRNA SLC16A1-AS1, lncRNA SLC7A11-AS1, lncRNA SNHG3, lncRNAs PITPNA-AS1, lncRNA PSMA3-AS1, lncRNA LINC00520, lncRNA HOXA11-AS, lncRNA GMDS-AS1, lncRNA SBF2-AS1, lncRNA SNHG16, lncRNA ZXF1, lncRNA FTX, lncRNA LUCAT1, lncRNA LINC00857, and lncRNA TM4SF1-AS1, respectively. **(F)** Clustering heatmap of the expression of 20 lncRNAs in exosomes from 20 plural effusion samples. Each column represents one sample and each row represents one lncRNA. Gene expression in shown in red (upregulation) and green (downregulation). ID B1-B5 represents 5 patients with benign disease. ID L1-L15 represents 15 patients with lung cancer. The data are expressed as mean ± SEM.

Exosomal PITPNA-AS1 was strikingly increased in the pleural effusions from lung cancer patients. Extracellular vesicles from the pleural effusions of 15 lung cancer patients (5 per with SCLC, LUAD, LUSC) and 5 non-cancer patients were isolated by ultracentrifugation, and they were demonstrated to be high-quality exosomes according to the following observations (8, 23, 24). Visualization using transmission electron microscopy (TEM) demonstrated that the extracellular vesicles were oval-shaped (**Figure 1A**). The Malvern Zetasizer Nano ZS90 system indicated that their diameters were 50 - 150 nm (**Figures 1B, C**). CD63 and CD9 expression was observed in the exosomes using western blotting (**Figure 1D**).

Accumulated evidence has revealed that exosomal lncRNAs regulate tumorigenesis. By reviewing the recent literature on cancer-associated lncRNAs, we selected 20 lncRNAs for further analysis, comprising lncRNA DLEU2, lncRNA UCA1, lncRNA CCAT2, lncRNA AC020978, lncRNA NNT-AS1, lncRNA SLC16A1-AS1, lncRNA SLC7A11-AS1, lncRNA SNHG3, lncRNA PITPNA-AS1, lncRNA PSMA3-AS1, lncRNA LINC00520, lncRNA HOXA11-AS, lncRNA GMDS-AS1, lncRNA SBF2-AS1, lncRNA SNHG16, lncRNA ZXF1, lncRNA FTX, lncRNA LUCAT1, lncRNA LINC00857, and lncRNA TM4SF1-AS1. The expression of these lncRNAs in exosomes from 20 plural effusion samples (from 15 lung cancer patients and 5 non-cancer patients) was shown in the clustering heatmap (**Figure 1D**). Based on the relative lncRNA expression level, the list of 20 lncRNAs of interest was narrowed down to 11 (**Figure 1F**). These 11 lncRNAs comprised lncRNA-AC020978, lncRNA NNT-AS1, lncRNA SLC16A1-AS1, lncRNAs PITPNA-AS1, lncRNA PSMA3-AS1, lncRNA LINC00520, lncRNA GMDS-AS1, lncRNA SBF2-AS1, lncRNA SNHG16, lncRNA LUCAT1, lncRNA LINC00857, and lncRNA TM4SF1-AS1, which were all significantly changed in the lung cancer patients compared to the non-cancer patients. Among these, the lncRNA PITPNA-AS1 had the highest significance and was remarkably increased in the lung cancer patients (**Figure 1E**). This lncRNA is smaller than the other lncRNAs and has gained more attention in the field of cancer research. Thus, the exosomal PITPNA-AS1 level was significantly and dramatically elevated in lung cancer, and it was subjected to further analysis.

### Exosomal PITPNA-AS1 in pleural effusions was positively associated with lung cancer pathological stage

**Figure 2:**
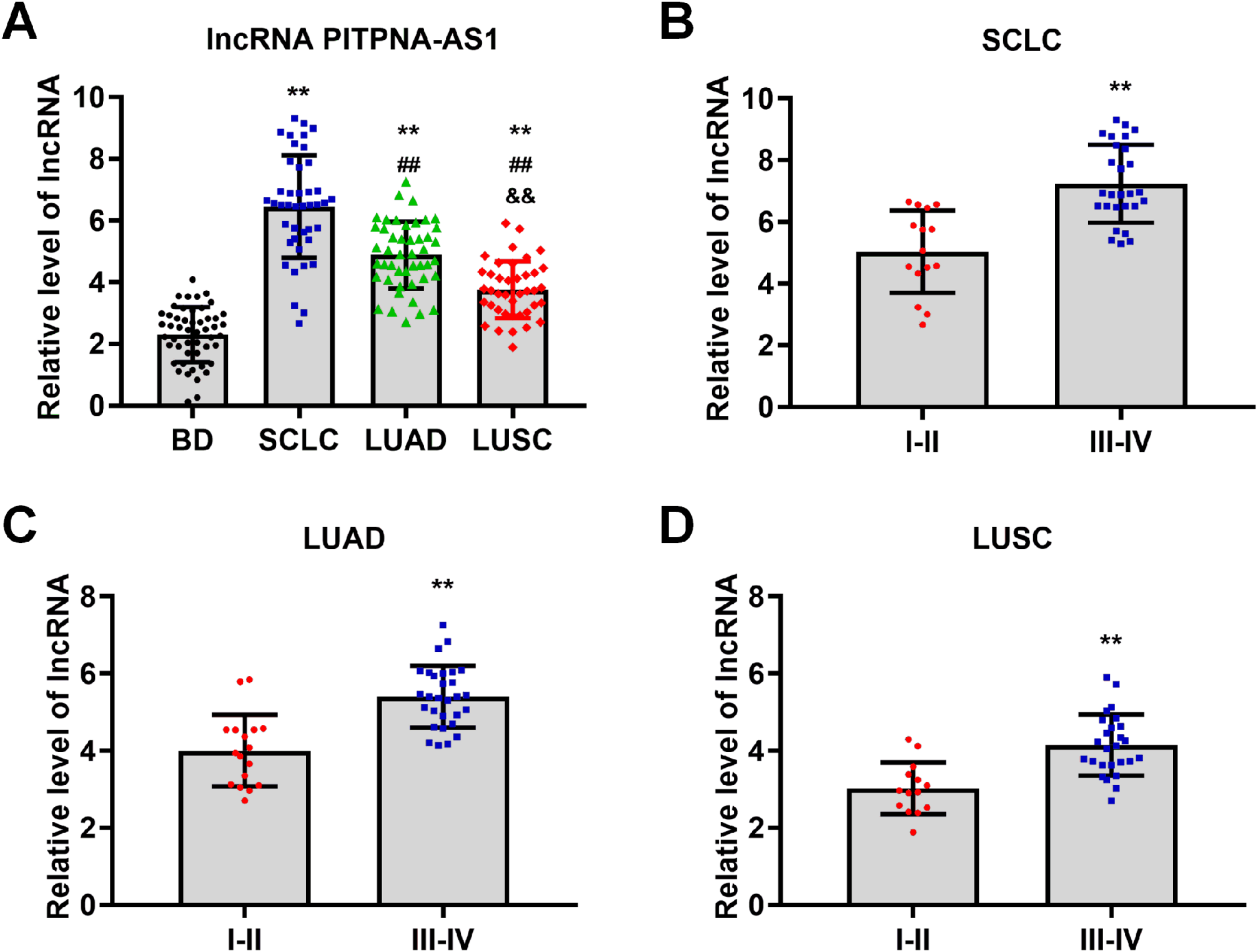
LncRNA PITPNA-AS1 is closely related to pathological stage in various lung cancer subtypes. **(A)** qRT-PCR analysis of lncRNA PITPNA-AS1 levels in 50 non-cancer patients, 42 SCLC patients, 46 LUAD patients, and 40 LUSC patients. **(B-D)** lncRNA PITPNA-AS1 levels in stage I–II and III–IV SCLC **(B)**, LUAD **(C),** and LUSC **(D)**. Statistical significance of between-group differences was determined by two-tailed Student’s t-tests. **p<0.01 vs. non-cancer cases or stage I–II. ##*p*<0.01 vs. SCLC. &&*p*<0.01 vs. LUAD.

To further evaluate the association between the exosomal PITPNA-AS1 level and lung cancer, we analyzed a validation cohort of 178 patients (50 non-cancer, 42 SCLC, 46 LUAD, and 40 LUSC patients). The quantitative comparison demonstrated that patients with the various lung cancer subtypes have higher exosomal PITPNA-AS1 levels than non-cancer patients (**Figure 2A**). Interestingly, there were significantly different levels among the patients with different lung cancer subtypes. Compared to non-cancer patients, the fold changes in patients with SCLC, LUAD, and LUSC were 2.80, 2.13, and 1.63, respectively. This suggests that the exosomal PITPNA-AS1 level might contribute to the pathological lung cancer subtype. On the basis of the medical records on tumor stage, patients with different lung cancer subtypes were divided into two groups, which represented the early (I–II) and advanced (III–IV) stages. We then investigated whether the exosomal PITPNA-AS1 level was associated with tumor stage in the lung cancer subtypes. Our analysis revealed that the exosomal PITPNA-AS1 level was lower at stage I–II than stage III–IV stage for SCLC, LUAD, and LUSC (all *p <*0.01, **Figures 2B–D**). The associations between exosomal PITPNA-AS1 levels and lung cancer clinicopathological features are presented in **Table 2**. For SCLC patients, exosomal lncRNA PITPNA-AS1 level was related to tumor stage, lymph node metastasis, and distant metastasis (all *p <*0.05). The rates of exosomal lncRNA PITPNA-AS1 positivity in SCLC, LUAD, or LUSC at stage III–IV were 65.5%, 71.0%, and 69.2%, respectively. Among the SCLC patients with high lncRNA PITPNA-AS1 levels, the proportions of patients with lymph node metastasis and distant metastasis were 90.9% and 54.5%, respectively, while the proportions of patients with low lncRNA PITPNA-AS1 levels were decreased, at 50.0% and 9.1%. Similar results were found for LUAD patients (91.7% and 33.3% vs. 54.2% and 4.2%, respectively) and LUSC patients (95.0% and 45.0% vs. 45.0% and 5.0%, respectively). Taken together, the data showed that high exosomal PITPNA-AS1 level in pleural effusion is positively related to lung cancer occurrence and advance lung cancer stage, which highlights the potential clinical significance of lncRNA PITPNA-AS1 in lung cancer diagnosis.

**Table 2:** The correlation between differentially expressed exo-PITPNA-AS1 and clinicopathological characteristics of patients with SCLC, LUAD and LUSC.

	SCLC			LUAD			LUSC		
Characteristic	H-lncRNA (n=22)	L-lncRNA (n=22)	P value	H-lncRNA (n=24)	L-lncRNA (n=24)	P value	H-lncRNA (n=20)	L-lncRNA (n=20)	P value
Gender (male/female)	14/8	15/7	0.500	15/9	13/11	0.385	8/12	6/14	0.371
Age (<65y≥65y)	16/6	18/4	0.360	10/14	9/15	0.500	10/10	9/11	0.500
Smoking story (Yes/No)	15/7	11/11	0.179	17/7	14/10	0.273	13/7	12/8	0.500
Stage (I-II/III-IV)	3/19	12/10	0.005	2/22	15/9	0.000	2/18	12/8	0.001
Lymph metastasis (Yes/No)	20/2	11/11	0.003	22/2	13/11	0.004	19/1	9/11	0.001
Distal metastasis (Yes/No)	12/10	2/20	0.001	8/16	1/23	0.011	9/11	1/19	0.004

According to the median of lncRNA PITPNA-AS1 in patients with different subtype, lung cancer patients were divided into two groups: L-lncRNA group and H-lncRNA group; SCLC, small cell lung cancer; LUAD, lung adenocarcinoma; LUSC, lung squamous cell carcinoma.

### Validation of exosomal PITPNA-AS1 indicated its independent diagnostic value in SCLC, LUAD, and LUSC

**Figure 3:**
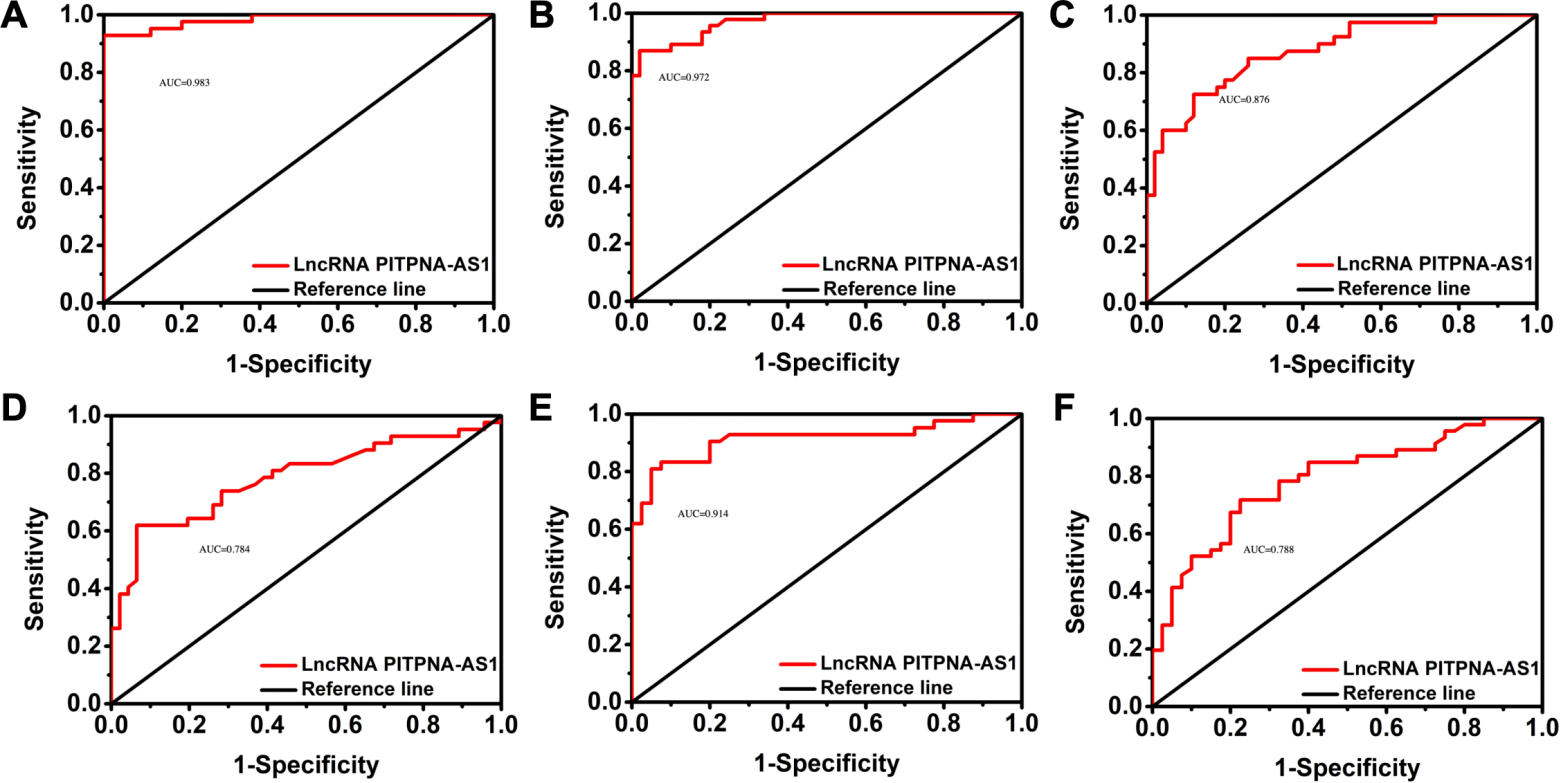
Validation of exosomal lncRNA PITPNA-AS1 level as a diagnostic biomarker in SCLC, LUAD, and LUSC. **(A-C)** ROC curves of exosomal lncRNA PITPNA-AS1 in non-cancer cases vs SCLC **(A)**, LUAD **(B)**, and LUSC **(C)**. **(D-F)** ROC curves of exosomal lncRNA PITPNA-AS1 in patients with SCLC, LUAD and LUSC.

Next, we verified the independent diagnostic value of exosomal PITPNA-AS1 in SCLC, LUAD, and LUSC. The 178 cases were subjected to receiver operating characteristic (ROC) curve analyses. The AUC for differentiating SCLC from non-cancer cases was 0.983, with a sensitivity of 92.9% and a specificity of 100% (**Figure 3A**, **Table 3**). The AUC for differentiating LUAD from non-cancer cases was 0.972, with a sensitivity of 87% and a specificity of 98% (**Figure 3B**, **Table 3**). The AUC for differentiating LUSC from non-cancer cases was 0.876, with a sensitivity of 85% and a specificity of 74% (**Figure 3C**, **Table 3**). Based on these results, we further explored the value of exosomal PITPNA-AS1 for distinguishing among SCLC, LUAD, and LUSC. The AUC for differentiating LUAD or LUSC from SCLC was 0.784 or 0.914, respectively, with a sensitivity of 61.9% and 81%, respectively, and a specificity of 93.5% and 95%, respectively (**Figures 3D, E**, **Table 3**). Moreover, the AUC for differentiating LUAD from LUSC was AUC of 0.788, with a sensitivity of 67.4% and a specificity of 80% (*p <*0.01; **Figure 3F**, **Table 3**). Our analysis strongly suggested that exosomal PITPNA-AS1 in pleural effusions could be used as an independent diagnostic biomarker in lung cancer subtypes.

**Table 3:** The data of ROC curve were corresponding to **Figure 3**, including AUC, 95% CI, sensitivity, specificity and Youden index.

Index	AUC	95%CI		P value	Sensitivity	Specificity	Yoden index	Critical value
		Down	UP					
SCLC vs BD	0.983	0.962	1	<0.01	92.9%	100%	0.929	4.215
LUAD vs BD	0.972	0.945	0.998	<0.01	87%	98%	0.850	3.65
LUSC vs BD	0.876	0.805	0.946	<0.01	85%	74%	0.59	2.87
SCLC vs LUAD	0.784	0.685	0.884	<0.01	61.9%	93.5%	0.554	6.275
SCLC vs LUSC	0.914	0.847	0.981	<0.01	81%	95%	0.76	5.215
LUAD vs LUSC	0.788	0.692	0.883	<0.01	67.4%	80%	0.474	4.5

BD, benign disease; SCLC, small cell lung cancer; LUAD, lung adenocarcinoma; LUSC, lung squamous cell carcinoma.

### PITPNA-AS1 acted as an oncogene to promoted proliferation and migration of lung cancer cells

**Figure 4:**
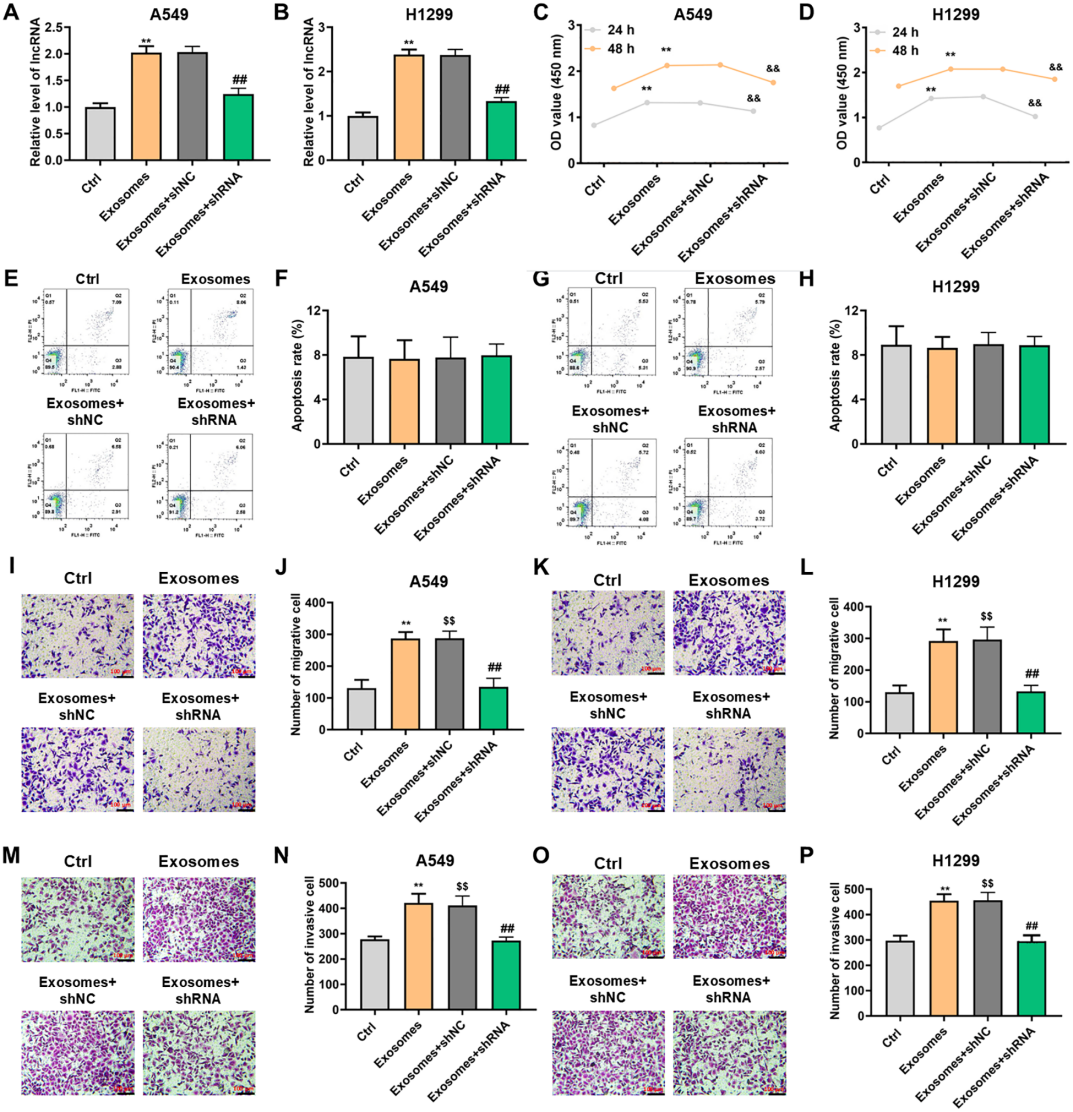
Upregulation or downregulation of exosomal lncRNA PITPNA-AS1 could promote or inhibit cell proliferation and motility *in vitro*. H1299 and A549 cells were pretreatment with shPITPNA-AS1 and then co-incubated with exosomes for the indicated times. **(A, B)** lncRNA PITPNA-AS1 expression assessed by qRT-PCR in A549 **(A)** and H1299 **(B)** cells. **(C, D)** Proliferation of A549 **(C)** and H1299 **(D)** cells assessed by CCK-8 assay. **(E, H)** Apoptosis of A549 **(E, F)** and H1299 **(G, H)** cells assessed by flow cytometry. **(I-P)** Cell invasion and migration assessed by transwell assay in A549 (left panel) and H1299 (right panel) cells. Scale bar, 100 μm. Statistical significance of between-group differences was determined by two-tailed Student’s t-tests. **p<0.01 vs. Ctrl with 24h. $$*p*<0.01 vs. Ctrl with 48h. &&*p*<0.01 vs. exosome+shNC with 24h. ##*p*<0.01 vs. exosome+shNC with 48h.

We next explored the biological function of lncRNA PITPNA-AS1 in H1299 and A549 lung cancer cells. qRT-PCR results illustrated that co-incubation with exosomes increased the relative lncRNA PITPNA-AS1 expression in H1299 and A549 cells, which indicated that the lncRNA PITPNA-AS1 enriched in exosomes could be transferred to lung cells (**Figures 4A, B**). To identify the specific lncRNA involved, we knocked down the PITPNA-AS1 expression with short hairpin RNAs (shRNA) in lung cells and the effect was identified (**Figures 4A, B**). Similarly, exosomes and lung cancer cells were co-incubated for 24 or 48 h. CCK-8 assays showed that this co-incubation significantly boosted cell proliferation, while the knockdown of PITPNA-AS1 repressed proliferation in A549 and H1299 (**Figures 4C, D**). Flow cytometry showed that there was no significant difference in apoptosis among those four groups (**Figures 4E–H**). These findings illustrated that PITPNA-AS1 upregulation or downregulation boosted or restrained the cell proliferation in lung cancer cells without affecting the cell apoptosis.

Next, we determined whether exosomal lncRNA PITPNA-AS1 contributes to cell invasion and migration in lung cancer. Transwell assays indicated that migration of H1299 or A549 cells was accelerated by exosomal PITPNA-AS1 co-incubation, and the phenomenon was neutralized through PITPNA-AS1 knockdown (**Figures 4I–L**). Transwell assays also showed that exosomal lncRNA PITPNA-AS1 increased the invasion ability of A549 cells, and vice versa (**Figures 4M, N**). Similar results were found in H1299 cells (**Figures 4O, P**). Taken together, these results confirmed that exosomal lncRNA PITPNA-AS1 co-incubation or PITPNA-AS1 knockdown promoted or suppressed the motility in lung cancer cells.

### FMR1 may be a functional target of PITPNA-AS1

**Figure 5:**
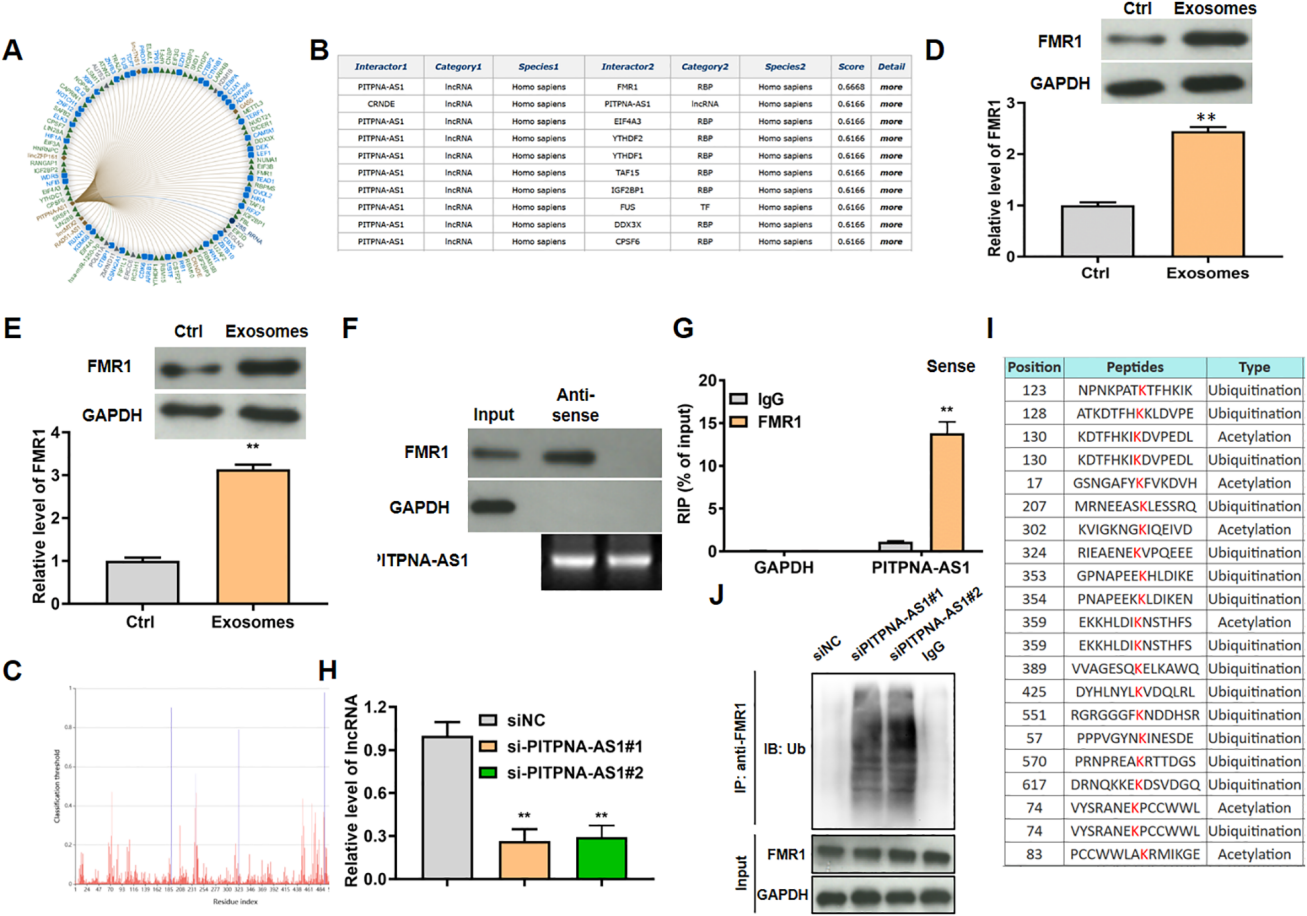
LncRNA PITPNA-AS1 modulated FMR1 ubiquitination *in vitro*. **(A)** Top 100 targeted genes predicted using the RNA Interactome Database. **(B)** The top 9 targeted genes with the confidence score. **(C)** The bind site of lncRNA PITPNA-AS1 on FMR1. **(D, E)** FMR1 expression in H1299 **(D)** and A549 **(E)** cells after co-incubation with exosomes from lung cancer patients. **(F)** Enrichment of PITPNA-AS1 and FMR1 assessed by RIP assay involving an anti-FMR1 antibody. **(G)** Interaction between lncRNA PITPNA-AS1 and FMR1 assessed by RNA pull-down assay. **(H)** FMR1 modification sites predicted using the Protein Lysine Modification Database (PLMD). **(I)** Relative lncRNA PITPNA-AS1 level after transfection with si-PITPNA-AS1#1/2. **(J)** Ubiquitination level of FMR1 after transfection with si-PITPNA-AS1. **p<0.01 vs. Ctrl or IgG.

Next, we predicted the targets of lncRNA PITPNA-AS1 in lung cells using the RNA Interactome Database (http://www.rna-society.org/rnainter/home.html). The top 100 predicted targets are shown in **Figure 5A**. Among these targets, lncRNA PITPNA-AS1 was most likely to bind to the protein FMR1 with highest score 0.6668 (**Figure 5B**). Based on this prediction, we assessed FMR1 expression in H1299 and A549 lung cancer cells after co-incubation with exosomes (containing PITPNA-AS1), and found that FMR1 protein expression was strikingly elevated (**Figures 5D, E**).

Mechanistic experiments were used to assess the interaction between FMR1 and lncRNA PITPNA-AS1 in A549 cells. In the RNA pull-down assays, FMR1 was pulled down by biotinylated lncRNA PITPNA-AS1 sense probe (**Figure 5F**). In the RIP assays, lncRNA PITPNA-AS1 was enriched in the material that was precipitated by anti-FMR1 antibody relative to nonspecific IgG (**Figure 5G**). By searching the Protein Lysine Modification Database (PLMD; http://plmd.biocuckoo.org/), we found that FMR1 (which is an RNA-binding protein) could be ubiquitinated at site 324, which is close to the RNA-binding site (site 323) of FMR1 (**Figures 5C, H**). Hence we tested the hypothesis that lncRNA PITPNA-AS1 regulated the level of FMR1 in ubiquitination-dependent manner. After knockdown of lncRNA PITPNA-AS1 in A549 cells using si-PITPNA-AS1#1 and si-PITPNA-AS1#2 (**Figure 5I**), RIP assays using anti-ubiquitin antibody were performed to assess FMR1 ubiquitination, showing that the levels of FMR1 ubiquitination were increased (**Figure 5J**). To sum up, lncRNA PITPNA-AS1 specifically bound to FMR1, which inhibited the ubiquitination of FMR1.

## Discussion

Accumulated evidence indicates that exosomes and their contents can serve as biomarkers for cancer diagnosis and prognosis (25, 26). Exosomes can be obtained from a variety of bodily fluids such as serum, breast milk, and ascites, and also pleural effusions (12, 24, 27, 28), and a large amount of pleural effusion can be obtained by minor access surgery. Thus, the use of isolated exosomes might be able to substitute surgical procedures for diagnosing lung cancer, as primary tumor tissues are difficult to obtain in most patients. In our study, we focused on the exosomes in pleural effusion, which might contain high-value valid biomarkers.

Exosomes may carry a broad range of lncRNAs and play critical roles in cancer occurrence and development by inhibiting translation or acting as competitive endogenous RNAs. The qRT-PCR analysis showed that 11 lncRNAs were particularly enriched in exosomes from lung cancer patients compared to non-cancer patients, while others were barely expressed, indicating that specific lncRNAs are selectively sorted into exosomes. The lncRNAs protected by specific proteins in exosomes might act as detectable biomarkers, though the additional underlying mechanisms have not yet been fully elucidated. In our study, we unexpectedly discovered that lncRNA PITPNA-AS1 was enriched in exosomes in pleural effusions from lung cancers. The exosomal lncRNA PITPNA-AS1 level in pleural effusions was highly significantly different between lung cancer and non-cancer patients (more significantly than the other differentially expressed lncRNAs). This is consistent with research by Sun et al. (29), in which PITPNA-AS1 was increased and functioned as an oncogene in HCC. Analysis also showed that exosomal PITPNA-AS1 was associated with tumor stage, lymph node metastasis, and distant metastasis. It is further suggested that exosomal PITPNA-AS1 may be helpful to differentiate among lung cancer subtypes. We found that not only did exosomal PITPNA-AS1 accurately distinguish SCLC patients from non-cancer patients with the highest AUC (0.983) and the highest sensitivity and specificity out of all the ROC curve analyses, but it also distinguished SCLC from LUSC very well, showing excellent sensitivity, specificity, and AUC value. Our findings provide an objective basis for distinguishing among lung cancer subtypes, and the lncRNA could also distinguish among tumor stages. The accuracy of using proteins combined with other biomarkers (such as PITPNA-AS1) to differentiate among lung cancer subtypes remains a research focus for the future.

LncRNAs such as PITPNA-AS1 are emerging as critical regulators of gene expression, participating in tumor progression and metastasis. It was reported that PITPNA-AS1 boosted LUSC cell proliferation and migration by recruiting TAF15 to stabilize HMGB3 (30). However, its exact effects on the progression of other lung cancer subtypes remain unknown. Through exosome co-incubation assays, we discovered that lncRNA PITPNA-AS1 can be delivered to cancer cells via exosomes. Gain-of-function and loss-of-function investigations found that lncRNA PITPNA-AS1 contributed to cell growth, invasion, and migration. This is consistent with the findings of Ren et al. (30). More importantly, lncRNA PITPNA-AS1 knockdown significantly inhibited the proliferation and migration of lung cancer cells, while co-incubation with exosomes could reverse this inhibition. However, co-incubation with exosomes did not significantly affect apoptosis, which may be due to the low baseline apoptosis level leading to no significant exosome-induced increase. further research on its function in malignant biological behaviors *in vivo* is also required.

Finally, we further explored the underlying mechanism of lncRNA PITPNA-AS1 regarding lung cancer progression. The database-based prediction of lncRNA PITPNA-AS1 targets indicated that Fragile X messenger ribonucleoprotein 1 (FMR1) had the highest confidence score. FMR1 is a RNA-binding protein and functions as a central regulator of transcription and translation in nervous system. Of note, emerging evidence indicates that FMR1 is increased in multiple cancers (31–33), such as colorectal cancer (34) and HCC (35). For example, Zalfa et al. (36) reported that FMR1 in metastatic melanoma cell lines affects cell migration, invasion, and adhesion. Therefore, we speculate that the involvement of lncRNA PITPNA-AS1 in the progression of lung cancer may be mediated by FMR1. In this study, the validated interaction between lncRNA PITPNA-AS1 and FMR1 in lung cancer was first presented, with lncRNA PITPNA-AS1 upregulation increasing FMR1 levels. Research has shown that the ubiquitin-proteasome pathway is a common endogenous protein degradation pathway. Takabatake et al. (37) found that this pathway is involved in Sema3A-induced FMR1 degradation in growth cones. Therefore, we investigated whether lncRNA PITPNA-AS1 affects FMR1 levels by regulating ubiquitination. We found that lncRNA PITPNA-AS1 binds to FMR1 at site 324, which might competitively hamper the approach of ubiquitination proteins to site 323. RIP assays confirmed that lncRNA PITPNA-AS1 knockdown increased the levels of FMR1 ubiquitination. These findings highlight the important effect of lncRNA PITPNA-AS1 on lung cancer progression by blocking FMR1 ubiquitination. However, the signaling pathway that mediates the inhibition of FMR1 ubiquitination by lncRNA PITPNA-AS1 remains unknown. Although FMR1 has been reported to be involved in cancer progression, an in-depth exploration of its biological function will contribute to understand the mechanism of lncRNA PITPNA-AS1-mediated proliferation and metastasis in lung cancer (38, 39).

Taking the results together, we showed that exosomal lncRNA PITPNA-AS1 is increased in pleural effusions from lung cancer patients. Furthermore, it had a high accuracy for differentiating among lung cancer subtypes. In addition, it was found to be involved in lung cancer progression by inhibiting FMR1 ubiquitination. Our study sheds a light on the potential use of exosomal lncRNA PITPNA-AS1 as an objective basis for the early diagnosis, early treatment, and prognosis of lung cancer.

## Data Availability

The datasets presented in this study can be found in online repositories. The names of the repository/repositories and accession number(s) can be found in the article/**Supplementary Material**.
