# Supplementary material for: Diagnostic value of exosome-derived lncRNA PITPNA-AS1 in lung cancer
Source: Front Immunol. 2025 Apr 24;16:1539557. doi: 10.3389/fimmu.2025.1539557 (PMC12058797; doi:10.3389/fimmu.2025.1539557)
Supplement: Supplementary file 1 [file DataSheet1.pdf]

Supplementary Material

1 Supplementary Figures and Tables

For more information on Supplementary Material and for details on the different file types accepted, please see here.

1.1 Supplementary Figures

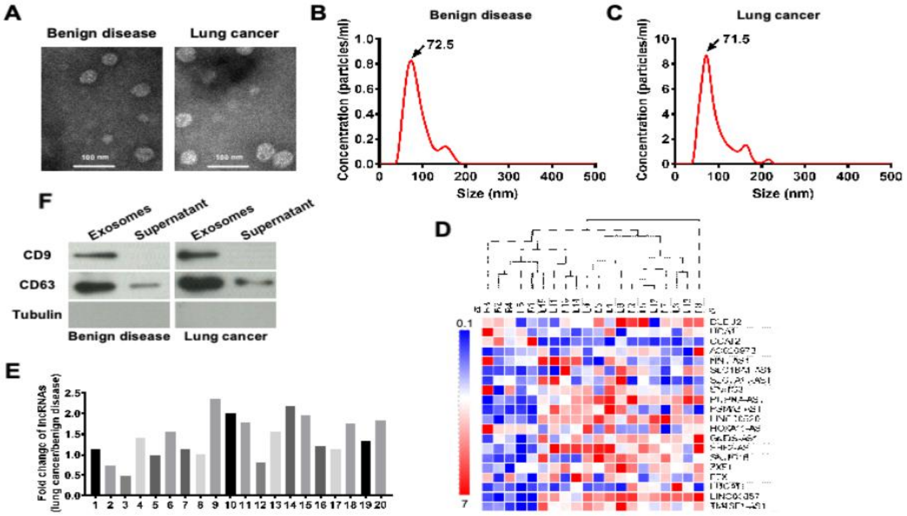

Figure 1. Exosomes were isolated from pleural effusions, and lncRNA PITPNA-AS1 was found to be enriched in the exosomes from lung cancer patients. **(A)** Representative transmisson electron microscopy images of exosomes from 15 lung cancers (5 per with SCLC, LUAD, LUSC) and 5 patients with benign disease. Scale bar: 100 nm. **(B, C)** Distribution of exosomes with diameters of 50–150 nm assessed using a Malvern Zetasizer Nano ZS90 system in benign disease group (B) and lung cancer group (C). **(D)** Level of indicated proteins in exosomes and supernatant from 15 lung cancers and 5 patients with benign disease. (E) Fold changes of lncRNA expression. The expression of each lncRNA in

benign disease group was normalized to 1. 1–20 indicates lncRNA DLEU2, lncRNA UCA1, lncRNA CCAT2, lncRNA-AC020978, lncRNA NNT-AS1, lncRNA SLC16A1-AS1, lncRNA SLC7A11-AS1, lncRNA SNHG3, lncRNAs PITPNA-AS1, lncRNA PSMA3-AS1, lncRNA LINC00520, lncRNA HOXA11-AS, lncRNA GMDS-AS1, lncRNA SBF2-AS1, lncRNA SNHG16, lncRNA ZXF1, lncRNA FTX, lncRNA LUCAT1, lncRNA LINC00857, and lncRNA TM4SF1-AS1, respectively. **(F)** Clustering heatmap of the expression of 20 lncRNAs in exosomes from 20 plural effusion samples. Each column represents one sample and each row represents one lncRNA. Gene expression in shown in red (upregulation) and green (downregulation). ID B1-B5 represents 5 patients with benign disease. ID L1-L15 represents 15 patients with lung cancer. The data are expressed as mean ± SEM.

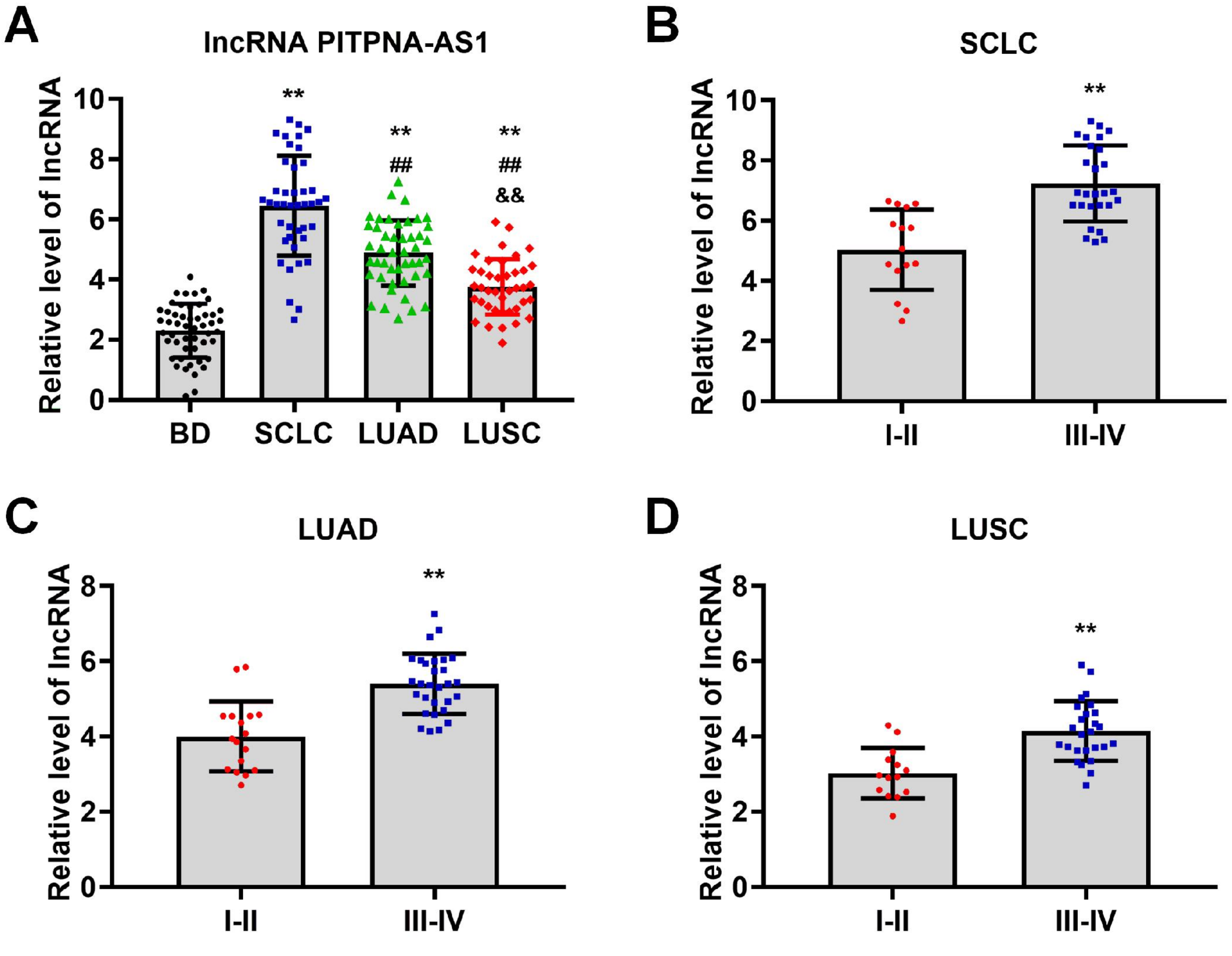

Figure 2. LncRNA PITPNA-AS1 is closely related to pathological stage in various lung cancer subtypes. **(A)** qRT-PCR analysis of lncRNA PITPNA-AS1 levels in 50 non-cancer patients, 42 SCLC patients, 46 LUAD patients, and 40 LUSC patients. **(B-D)** lncRNA PITPNA-AS1 levels in stage I–II and III–IV SCLC **(B)**, LUAD **(C),** and LUSC **(D)**. Statistical

significance of between-group differences was determined by two-tailed Student's t-tests. **, p<0.01 vs. non-cancer cases or stage I–II. ##, p<0.01 vs. SCLC. &&, p<0.01 vs. LUAD.

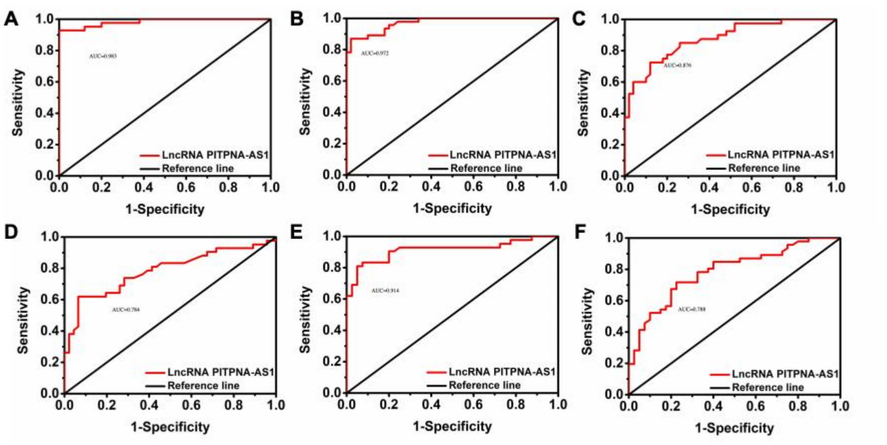

Figure 3. Validation of exosomal lncRNA PITPNA-AS1 level as a diagnostic biomarker in SCLC, LUAD, and LUSC. **(A-C)** ROC curves of exosomal lncRNA PITPNA-AS1 in non-cancer cases vs SCLC **(A)**, LUAD **(B)**, and LUSC **(C)**. **(D-F)** ROC curves of exosomal lncRNA PITPNA-AS1 in patients with SCLC, LUAD and LUSC.

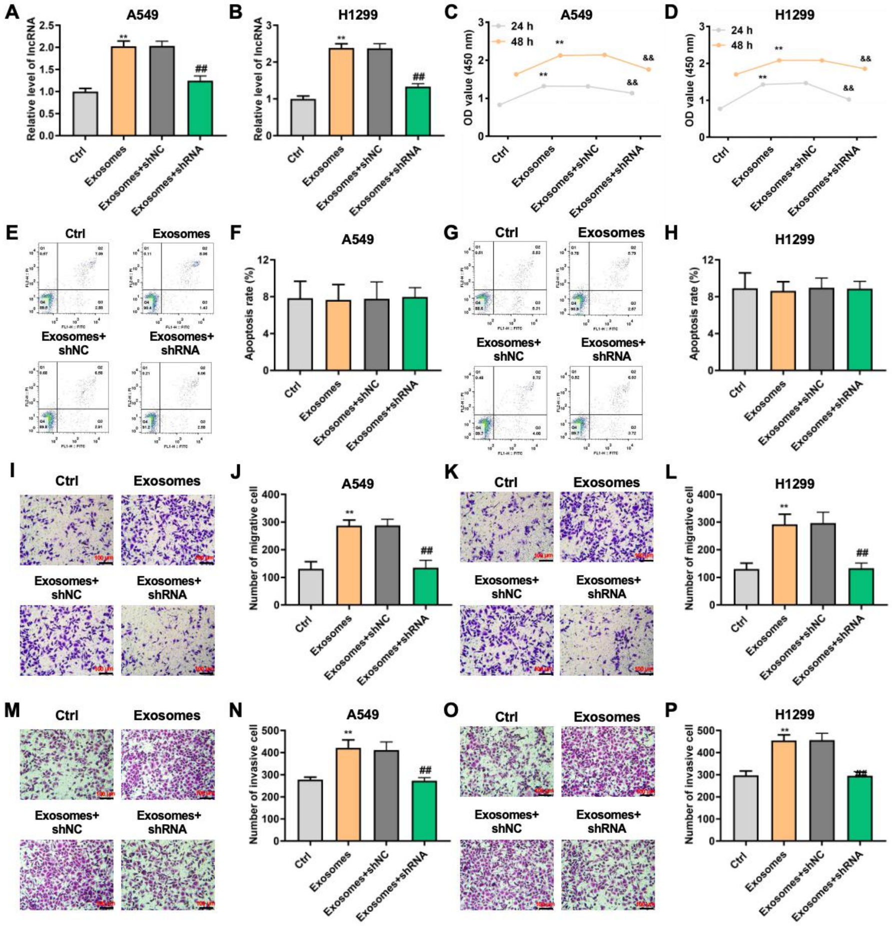

Figure 4. Upregulation or downregulation of exosomal lncRNA PITPNA-AS1 could promote or inhibit cell proliferation and motility *in vitro*. H1299 and A549 cells were pretreatment with shPITPNA-AS1 and then co-incubated with exosomes for the indicated times. **(A-B)** lncRNA PITPNA-AS1 expression assessed by qRT-PCR in A549 **(A)** and H1299 **(B)** cells. **(C-D)** Proliferation of A549 **(C)** and H1299 **(D)** cells assessed by CCK-8 assay. **(E-H)** Apoptosis of A549 **(E, F)** and H1299 **(G, H)** cells assessed by flow cytometry. **(I-P)** Cell invasion and migration assessed by transwell assay in A549 (left panel) and H1299 (right panel) cells. Scale bar, 100 μm. Statistical significance of between-group

differences was determined by two-tailed Student's t-tests. **, *p*<0.01 vs. Ctrl with 24h. $$, *p*<0.01 vs. Ctrl with 48h. &&, *p*<0.01 vs. exosome+shNC with 24h. ##, *p*<0.01 vs. exosome+shNC with 48h.

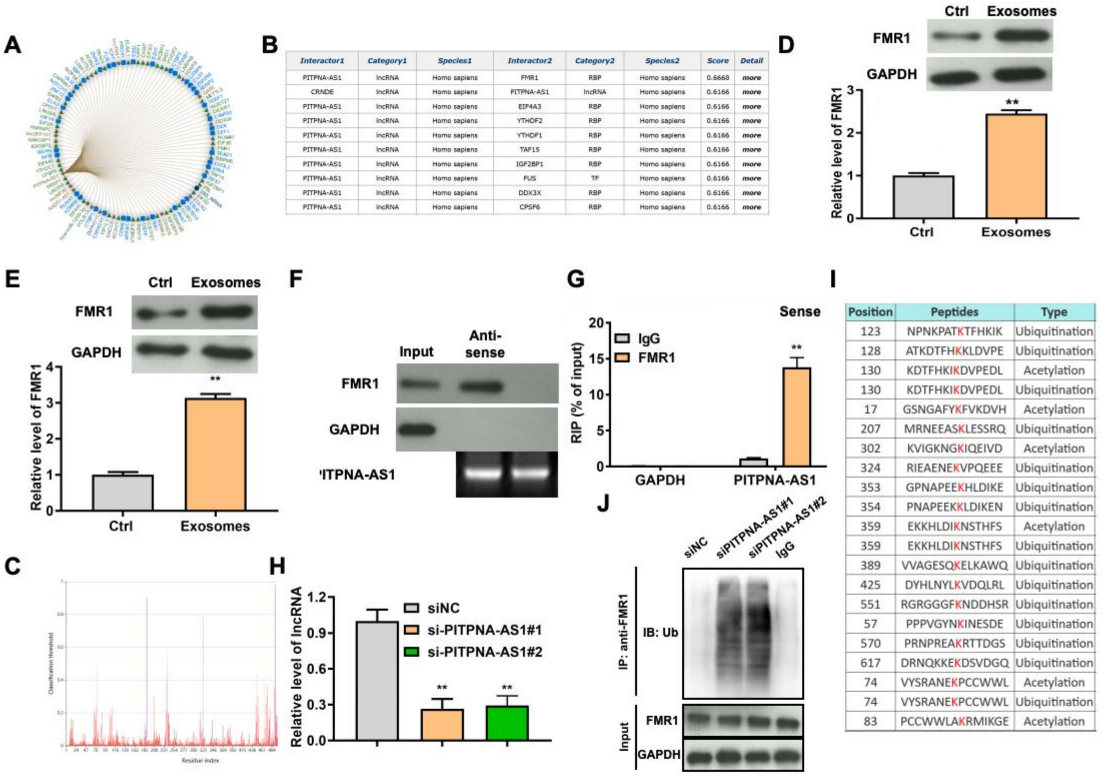

Figure 5. LncRNA PITPNA-AS1 modulated FMR1 ubiquitination *in vitro*. **(A)** Top 100 targeted genes predicted using the RNA Interactome Database. (B) The top 9 targeted genes with the confidence score. (C) The bind site of lncRNA PITPNA-AS1 on FMR1. **(D-E)** FMR1 expression in H1299 **(D)** and A549 **(E)** cells after co-incubation with exosomes from lung cancer patients. **(F)** Enrichment of PITPNA-AS1 and FMR1 assessed by RIP assay involving an anti-FMR1 antibody. **(G)** Interaction between lncRNA PITPNA-AS1 and FMR1 assessed by RNA pull-down assay. **(H)** FMR1 modification sites predicted using the Protein Lysine Modification Database (PLMD). **(I)** Relative lncRNA PITPNA-AS1

level after transfection with si-PITPNA-AS1#1/2. **(J)** Ubiquitination level of FMR1 after transfection with si-PITPNA-AS1. **, $p<0.01$ vs. Ctrl or IgG.

Table S1 Primer sequences of products expression

Gene name	Primer name	primer sequence (5'-3')
lncRNA DLEU2	Forward	TCTGGAGAACAGCCTCACTTC
	Reverse	TGCTGAGCTAAGTAGAGGTCTC
lncRNA UCA1	Forward	CTCTCCATTGGGTTCAC
	Reverse	GCGGCAGGTCTTAAGAGATGAG
lncRNA CCAT2	Forward	GCGCTGACAGAGATTGCTTAC
	Reverse	CCAGAGTAGAACAGGGGAAGC
lncRNA -AC020978	Forward	GGACCATGCAGTTTTGTGGAAGA
	Reverse	GGCAGAGGCAGGTGGATCAC
lncRNA NNT-AS1	Forward	CTGGAATCCCTGCTACTCAGGA
	Reverse	GCCATGTGATATGCCTGCTC
lncRNA SLC16A1-AS1	Forward	TGGACGATGCATATGTGGGG
	Reverse	CACGTTGGTTATGCGGTCAC.
lncRNA SLC7A11-AS1	Forward	CATCCTGTGGCTGGAGAGAT
	Reverse	AGCACTCGGAAAATGGTGAA

lncRNA SNHG3	Forward	TTCCGGGCGTTACTTAAGG
	Reverse	GGTCAAGAACAAGCACACCAA
lncRNA PITPNA-AS1	Forward	GCAGGGTGGATAAAGAGGA
	Reverse	CCTACTGACAGGATGTCCT
lncRNA PSMA3-AS1	Forward	GUCGGUCAGGUUGGUGUCUA
	Reverse	GCUGUGAAAGUGCCUGUGAA
lncRNA LINC00520	Forward	GTGCTAGGAGGACCCATACG
	Reverse	GCCACCAATGTGCTGTCAAG
lncRNA HOXA11-AS	Forward	TGCCAAGTTGTACTTACTACGTC
	Reverse	GTTGGAGGAGTAGGAGTATGTA
lncRNA GMDS-AS1	Forward	TGCTTT GAGGCCAAGCTA
	Reverse	TGGGTTCATAAGGGTTGCAT
lncRNA SBF2-AS1	Forward	CAGAAGGAGUCUACUGCUAAG
	Reverse	UAGCAGUAGACUCCUUCUGGG
lncRNA SNHG16	Forward	CAGTCAGCCTCAGTTTCCAA

	Reverse	AGGCAGGGCTGTGCTGAT
lncRNA ZXF1	Forward	GCCUGGUGGUAAAUAUGAATT
	Reverse	UUCAUAUUUACCACCAGGCTT
lncRNA FTX	Forward	GTGTCTCTCTCTCTCTCTCTT
	Reverse	CCTCTTCAGCAGTAGCATAGTT
lncRNA LUCAT1	Forward	AAGGTCCATATTAAACGTCCTACAA
	Reverse	TAGCCATTAGACTGCCAGAGGA
lncRNA LINC00857	Forward	CCCCTGCTTCATTGTTTCCC
	Reverse	AGCTTGTCCTTCTTGGGTACT
lncRNA TM4SF1-AS1	Forward	TGCAAGTCACTCTGATGCCG
	Reverse	AGCTCTGAGCAAACCATCCTC
GAPDH	Forward	GAAGGTGAAGGTCGGAGT
	Reverse	GAAGATGGTGATGGGATTTC

Table 1 Patient characteristics

Characteristic	Benign	Lung Cancer

	Disease (n=50)	SCLC (n=44)	LUAD (n=48)	LUSC (n=40)
Age in years Median (range)	62 (57-67.25)	61.5 (57-64)	66.5 (60.25-71)	65 (59.25-68)
Gender				
Male	33 (66%)	29 (65.91%)	28 (58.33%)	14 (35%)
Female	17 (34%)	15 (34.09%)	20 (41.67%)	26 (65%)
Smoking status				
Yes	29 (58%)	26 (59.09%)	31 (64.58%)	25 (62.5%)
No	21 (42%)	18 (40.91%)	17 (35.42%)	15 (37.5%)
Stage				
I-II	/	15 (34.09)	17 (35.42%)	14 (35%)
III-IV	/	29 (55.91%)	31 (54.58%)	26 (65%)
Lymph metastasis				
Yes	/	31 (70.45%)	25 (50.83%)	28 (70%)
No	/	13 (28.55%)	13 (49.17%)	12 (30%)
Distal metastasis				
Yes	/	14 (31.82%)	9 (18.75%)	10 (25%)
No	/	30 (58.18%)	39 (81.25%)	30 (75%)

ECOG, Eastern cooperative oncology group. SCLC, small cell lung cancer. LUAD, lung adenocarcinoma. LUSC, lung squamous cell carcinoma

Table 2 The correlation between differentially expressed exo-PITPNA-AS1 and clinicopathological characteristics of patients with SCLC, LUAD and LUSC.

Characteristic	SCLC			LUAD			LUSC		
	H-lncRNA (n=22)	L-lncRNA (n=22)	P value	H-lncRNA (n=24)	L-lncRNA (n=24)	P value	H-lncRNA (n=20)	L-lncRNA (n=20)	P value
Gender (male/female)	14/8	15/7	0.500	15/9	13/11	0.385	8/12	6/14	0.371
Age (<65y≥65y)	16/6	18/4	0.360	10/14	9/15	0.500	10/10	9/11	0.500
Smoking story (Yes/No)	15/7	11/11	0.179	17/7	14/10	0.273	13/7	12/8	0.500
Stage (I-II/III-IV)	3/19	12/10	0.005	2/22	15/9	0.000	2/18	12/8	0.001
Lymph metastasis (Yes/No)	20/2	11/11	0.003	22/2	13/11	0.004	19/1	9/11	0.001
Distal metastasis (Yes/No)	12/10	2/20	0.001	8/16	1/23	0.011	9/11	1/19	0.004

According to the median of lncRNA PITPNA-AS1 in patients with different subtype, lung cancer patients were divided into two groups: L-lncRNA group and H-lncRNA group. SCLC, small cell lung cancer. LUAD, lung adenocarcinoma. LUSC, lung squamous cell carcinoma.

Table 3 The data of ROC curve were corresponding to figure 3, including AUC, 95% CI, sensitivity, specificity and Youden index.

Index	AUC	95%CI	P value	Sensitivity	Specificity	Yoden index	critical value

		Down	UP					
SCLC vs BD	0.983	0.962	1	< 0.01	92.9%	100%	0.929	4.215
LUAD vs BD	0.972	0.945	0.998	< 0.01	87%	98%	0.850	3.65
LUSC vs BD	0.876	0.805	0.946	< 0.01	85%	74%	0.59	2.87
SCLC vs LUAD	0.784	0.685	0.884	< 0.01	61.9%	93.5%	0.554	6.275
SCLC vs LUSC	0.914	0.847	0.981	< 0.01	81%	95%	0.76	5.215
LUAD vs LUSC	0.788	0.692	0.883	< 0.01	67.4%	80%	0.474	4.5

BD, benign disease. SCLC, small cell lung cancer. LUAD, lung adenocarcinoma. LUSC, lung squamous cell carcinoma.
